# Cyclic Nitroxide 4-Methoxy-Tempo May Decrease Serum Amyloid A-Mediated Renal Fibrosis and Reorganise Collagen Networks in Aortic Plaque

**DOI:** 10.3390/ijms25147863

**Published:** 2024-07-18

**Authors:** Antony Gao, Kangzhe Xie, Sameesh Gupta, Gulfam Ahmad, Paul K. Witting

**Affiliations:** Redox Biology Group, Discipline of Pathology, Faculty of Medicine and Health, Charles Perkins Centre, The University of Sydney, Sydney, NSW 2006, Australia; agao2399@uni.sydney.edu.au (A.G.); kangzhe.xie@sydney.edu.au (K.X.); sameesh.gupta@anu.edu.au (S.G.); gulfam.ahmad@rch.org.au (G.A.)

**Keywords:** serum amyloid A, kidney dysfunction, atherosclerosis, pro-inflammation

## Abstract

Acute-phase serum amyloid A (SAA) can disrupt vascular homeostasis and is elevated in subjects with diabetes, cardiovascular disease, and rheumatoid arthritis. Cyclic nitroxides (e.g., Tempo) are a class of piperidines that inhibit oxidative stress and inflammation. This study examined whether 4-methoxy-Tempo (4-MetT) inhibits SAA-mediated vascular and renal dysfunction. Acetylcholine-mediated vascular relaxation and aortic guanosine-3′,5′-cyclic monophosphate (cGMP) levels both diminished in the presence of SAA. 4-MetT dose-dependently restored vascular function with corresponding increases in cGMP. Next, male ApoE-deficient mice were administered a vehicle (control, 100 µL PBS) or recombinant SAA (100 µL, 120 µg/mL) ± 4-MetT (at 15 mg/kg body weight via i.p. injection) with the nitroxide administered before (prophylaxis) or after (therapeutic) SAA. Kidney and hearts were harvested at 4 or 16 weeks post SAA administration. Renal inflammation increased 4 weeks after SAA treatment, as judged by the upregulation of IFN-*γ* and concomitant increases in iNOS, p38MAPK, and matrix metalloproteinase (MMP) activities and increased renal fibrosis (Picrosirius red staining) in the same kidneys. Aortic root lesions assessed at 16 weeks revealed that SAA enhanced lesion size (vs. control; *p* < 0.05), with plaque presenting with a diffuse fibrous cap (compared to the corresponding aortic root from control and 4-MetT groups). The extent of renal dysfunction and aortic lesion size was largely unchanged in 4-MetT-supplemented mice, although renal fibrosis diminished at 16 weeks, and aortic lesions presented with redistributed collagen networks. These outcomes indicate that SAA stimulates renal dysfunction through promoting the IFN-*γ*–iNOS–p38MAPK axis, manifesting as renal damage and enhanced atherosclerotic lesions, while supplementation with 4-MetT only affected some of these pathological changes.

## 1. Introduction

An intact vascular endothelium serves as barrier between circulating blood components and the major organs to which vessels supply oxygenated (and remove deoxygenated) blood under physiological conditions [1]. Various pathological stimuli alter the endothelium to promote an activated inflammatory phenotype that manifests as an altered vascular tone and a pro-thrombotic surface that promotes leukocyte recruitment to the inflamed vessel, and along with oxidative stress promote a heightened pathophysiological state [2]. Thus, endothelial dysfunction is considered an early event linked to vascular pathologies, preceding chronic inflammatory disorders such as cardiovascular disease [3,4].

Circulating levels of the hepatic acute-phase apolipoprotein serum amyloid A (SAA) increase rapidly in response to pro-inflammatory stimuli. Circulating SAA can increase markedly up to 1000-fold above normal physiological levels [5], and persistently elevated SAA levels are common in chronic disease states such as inflammatory bowel disease and rheumatoid arthritis [6,7,8]. SAA can be sequestered to high-density lipoproteins (HDLs) upon the release of mature particles into the circulation. Unbound SAA, which represents a fraction of the total circulating levels, can increase systemic inflammation through the interaction of the acute-phase protein with the endothelium [9,10] and peripheral blood mononuclear [11,12] cells. Furthermore, extrahepatic synthesis increases SAA levels in atherosclerotic lesions through expression by vascular smooth muscle cells and activated macrophages within the developing neointimal lesion [13,14].

The biological activity of SAA is linked to the transcriptional activation of nuclear factor kappa-light-chain-enhancer of activated B-cell (NF-κB) through both receptor- and ROS-mediated molecular pathways [15,16]. Therefore, SAA elicits inflammation via NF-κB signalling including T-cell activation, which is further linked to the production of interferon gamma (IFN-*γ*) and concomitant increases in inducible nitric oxide synthase (iNOS) [17,18,19]. This complex inflammatory milieu can then stimulate a cytokine cascade including tumour necrosis factor alpha (TNF-α) and pro-inflammatory interleukins (IL), which together act to potentiate the tissue inflammatory response to circulating SAA [20,21]. Activated macrophages also yield a variety of oxidants that cause localised oxidative stress, which further activates NF-κB in a cyclic loop, again exacerbating SAA-mediated inflammation leading to p38MAP-kinase (MAPK) activation and subsequent increases in tissue fibrosis in chronically inflamed tissues [9,16].

Cyclic nitroxides are a class of antioxidants with multiple biological activities. For example, cyclic nitroxides attenuate the severity of asthma-related inflammation and pulmonary fibrosis [22,23] and are credited with inhibiting radiation damage to in vitro cell cultures and biological tissues [24,25,26]. In addition, cyclic nitroxides have some therapeutic potential in the treatment of cancer through limiting ROS-stimulated tumorigenesis [27,28,29]. Cyclic nitroxides also ameliorate acute ischemia–reperfusion injury during experimental heart attack and stroke, purportedly via inhibiting ROS-mediated damage after acute arterial occlusion [30]. In the context of endothelial dysfunction (an early event in the process of atherogenesis), the cyclic nitroxide 4-methoxy-tempo (4-MetT) inhibits the SAA-mediated activation of cultured aortic endothelial cells, subsequently decreasing the adhesion of immune cells [31], suggestive of an anti-atherosclerotic action.

The primary aim of this study is to investigate whether SAA-mediated renal and vascular dysfunction can be ameliorated with 4-metT administered either before (prophylaxis) or after (therapeutic) supplementing recombinant SAA in atherosclerosis-prone apolipoprotein E-deficient (ApoE^−/−^) mice. Given the previous in vitro data which demonstrated the atheroprotective activity of 4-MetT in the context of SAA-mediated endothelial dysfunction, it is hypothesised that, in vivo, prophylactic or therapeutic supplementation of 4-MetT may mitigate pro-oxidative and pro-inflammatory histological markers induced by SAA and therefore may potentially offer renal and cardioprotective benefits. We note that this intervention study represents an extension of the previously published study [32] that was performed concomitantly and investigated the effects of supplementing recombinant SAA in ApoE^−/−^ mice.

## 2. Results

### 2.1. The Cyclic Nitroxide 4-MetT Inhibits SAA-Mediated Endothelial Dysfunction in Isolated Aortae

**Figure 1:**
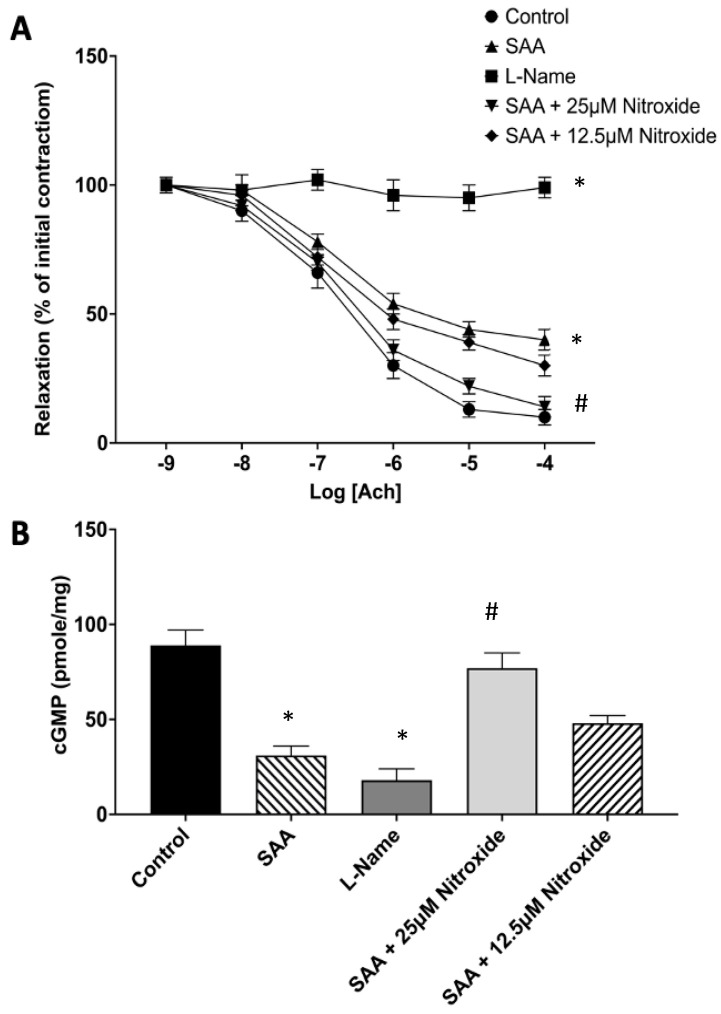
Vascular reactivity and cGMP level in response to SAA and nitroxide treatments. Isolated rat aortic rings were mounted into an 37 °C aerated Myobath system with 5% *v*/*v* CO_2(g)_. Individual aortae were treated with SAA supplemented with 50 mM PBS (Control), 10 µg/mL SAA (SAA), 20 µM L-Name (L-Name; eNOS inhibitor), 10 µg/mL SAA + 25 µM 4-MetT (SAA + 25 µM nitroxide), or 10 µg/mL SAA + 12.5 µM of 4-MetT (SAA + 12.5 µM nitroxide). (**A**) The presence of SAA decreased vascular reactivity as judged by the inhibition of acetylcholine (Ach)-induced endothelial-dependent relaxation. Pre-treatment with 4-MetT improved vascular response in a weakly dose-dependent manner. (**B**) The administration of SAA significantly reduced aortic cGMP levels, whilst supplementation with 4-MetT restored normal levels of cGMP in a dose-dependent manner. Data are expressed as relative means ± SD, *n* = 6 aortic rings per treatment group. * Different from the control group; *p* < 0.0001; ^#^ different from the SAA treatment group; *p* < 0.0001.

Preconstructed aortic segments relaxed completely with an increasing dose of the endothelium-dependent vasostimulator Ach, achieving ~95% of maximal relaxation (Figure 1). As anticipated, vascular relaxation was completely inhibited by all doses of Ach in the presence of L-NAME (positive control). In vessels treated with SAA alone, aortic relaxation was significantly impaired and only achieved ~60% relaxation to the highest dose of Ach tested. In the presence of the cyclic nitroxide 4-MetT, the SAA-mediated inhibition of aortic relaxation was ameliorated, and this was significant at the highest dose of nitroxide tested. As anticipated, the evaluation of aortic cGMP at the highest dose of Ach tested reflected the degree of relaxation determined in the same aortic samples, with cGMP levels significantly decreasing in the presence of SAA and L-NAME, while cotreatment with SAA and cyclic nitroxide (at 25 μM) restored aortic cGMP. Therefore, 4-MetT reversed SAA-mediated vascular dysfunction assessed ex vivo, and we next investigated the impact of cyclic nitroxide intervention in vivo.

### 2.2. SAA Stimulates MMP Activity in Mouse Kidneys in the Absence or Presence of 4-MetT

**Figure 2:**
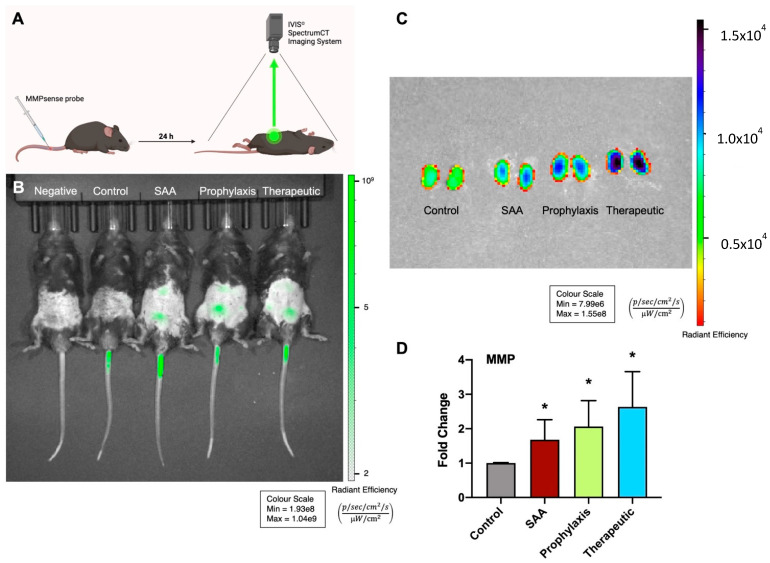
Assessment of MMP activity. Male ApoE^−/−^ mice were randomly allocated into 4 groups as detailed in the Methods section. (**A**) At 4 weeks post-treatment, mice were administered with 2 nmol MMPsense probe via tail venous injection. Next, animals were imaged with IVIS^®^ SpectrumCT while under sedation (green arrow in schematic represents bioluminescence signal). (**B**) Qualitative live animal imaging revealed trends of elevated bioluminescent signalling within the medial aspects of the lower thoracic and lower abdominal regions, corresponding with increased renal MMP activity, in the SAA, prophylaxis, and therapeutic groups compared with the untreated and vehicle-treated controls. Note, MMP activity shown in the tail section was localised to the entry point of tail-vein injection. (**C**) Isolated kidneys were reimaged and exhibited enhanced MMP bioluminescent signals in the SAA, prophylaxis, and therapeutic groups relative to the vehicle control mice. (**D**) Quantification of MMP bioluminescent signals from the isolated kidneys revealed significantly elevated (2–3-fold) MMP activity in the SAA, prophylaxis, and therapeutic treatment groups relative to the vehicle control mice. Data are expressed as fold-change vs. control (mean ± SD); different from the control * *p* ≤ 0.05. Note, the data pertaining to the control and SAA-treated mice shown in this figure were previously published in a truncated form [32]. The nitroxide intervention was conducted contemporaneously and is now compared to the same published data from the vehicle control and SAA-treated mice. Schematic in panel (**A**) was generated using BioRender.com under licence from the publisher.

In the atherosclerosis-prone ApoE^−/−^ mice, treatment with SAA stimulated an increase in renal MMP activity, as demonstrated by visualising the MMPSense bioprobe (Figure 2B), as judged by comparing bioluminescence in the lower abdominal region of naïve mice and mice treated with the probe via tail vein injection. Mice treated with 4-MetT prior to/or after SAA administration showed similar bioluminescence intensities in the lower abdominal region 24 h after introducing the MMPSense probe. Consistent with the in vivo imaging outcomes, the imaging of excised kidneys from the control, SAA, and 4-MetT mice indicated enhanced signals for all treatments (Figure 2C) that were significantly greater than those of the control (Figure 2D), but not different from each other.

### 2.3. SAA Elicits Increased Expression of KIM-1 and Total Urinary Protein in the Absence or Presence of 4-MetT

**Figure 3:**
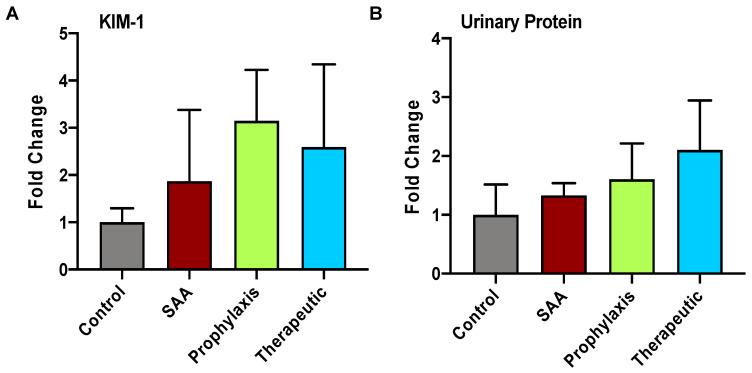
Measurement of acute markers of renal injury and renal dysfunction. Male ApoE^−/−^ mice were randomly allocated into 4 groups as detailed in the Methods section. Four weeks after the cessation of treatment, renal tissue allocated for biochemical analyses was harvested and homogenised and assessed for (**A**) KIM-1 by means of ELISA according to the manufacturer’s instructions. (**B**) Total urinary protein content was assessed using the bicinchoninic acid assay. Data are expressed as relative means ± SD. Note, the data pertaining to the control and SAA-treated mice shown in this figure were previously published in a truncated form [32]. The nitroxide intervention was conducted contemporaneously and is now compared to the same published data from the vehicle control and SAA-treated mice.

The data shown in Figure 2 indicate a likelihood that SAA elicits inflammation leading to accumulation of active MMPs in the renal tissue of SAA-treated mice. To evaluate whether the same kidneys showed evidence of renal dysfunction, the urinary levels of KIM-1 (maker of renal tubule injury) and total protein contents were determined (Figure 3). These data showed trends regarding increased KIM-1 excretion, but these changes were not significantly different from the vehicle control mice in the absence of SAA. Also, both markers of renal damage increased marginally in mice treated with 4-MetT, although again this was not significantly different from SAA (alone).

### 2.4. SAA Stimulates Renal Tubule Epithelium Expression of the Transcription Factor Nuclear Factor Erythroid 2-Related Factor 2 Independent of the Presence or Absence of 4-MetT

**Figure 4:**
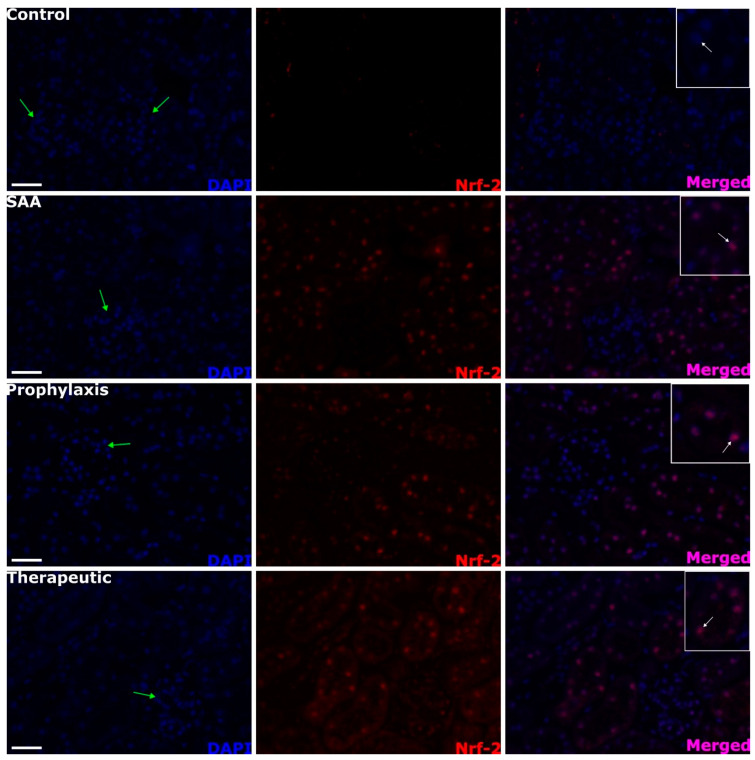
Renal antioxidative Nrf-2^+^ immunostaining patterns within the cortical region. Male ApoE^−/−^ mice were randomly allocated into 4 groups as detailed in the Methods section. Four weeks after the cessation of treatment, renal tissue was allocated for histological analyses. Patterns of cortical Nrf-2^+^ expression were assessed using immunofluorescence microscopy at 40× magnification (scale bar = 20 µm). Images are representative of at least 4 fields of view captured for each tissue section from *n* = 6 mice. Cell nuclei were stained with DAPI (blue) and Nrf-2 expression with a relevant fluorophore (red), or merged (purple). Green arrows indicate the location of glomeruli within the renal cortex, which exhibited scant Nrf-2^+^ immunostaining. White arrows (insets) point out relatively high nuclear Nrf-2^+^ immunostaining within tubular epithelial cells. Note, the data pertaining to the control and SAA-treated mice shown in this figure were previously published in a truncated form [32]. The nitroxide intervention was conducted contemporaneously and is now compared to the same published data from the vehicle control and SAA-treated mice.

SAA treatment stimulated the increased expression of Nrf-2 in the renal cortex, which was absent in the vehicle-treated control mice (c.f., control and SAA panels in Figure 4). This altered protein expression was localised to the proximal tubule epithelial cells in SAA-treated mice (see regions identified by white arrows in the merged images), while cells comprising the glomeruli (that is, the parietal epithelial cells that define the Bowman’s capsule and the cells within the glomerular tuft) showed no expression of Nrf-2 (refer to the glomeruli identified by the cluster of DAPI^+^-stained nuclei marked with green arrows). The extent and localisation of Nrf-2 expression were not altered by 4-MetT administered either before or after SAA treatment (c.f., SAA panel with 4-MetT administered in prophylaxis or therapeutic interventions, Figure 4).

### 2.5. SAA Stimulates Phospho-NF-kB p-65 Expression in the Renal Tissue Independent of the Presence or Absence of 4-MetT

**Figure 5:**
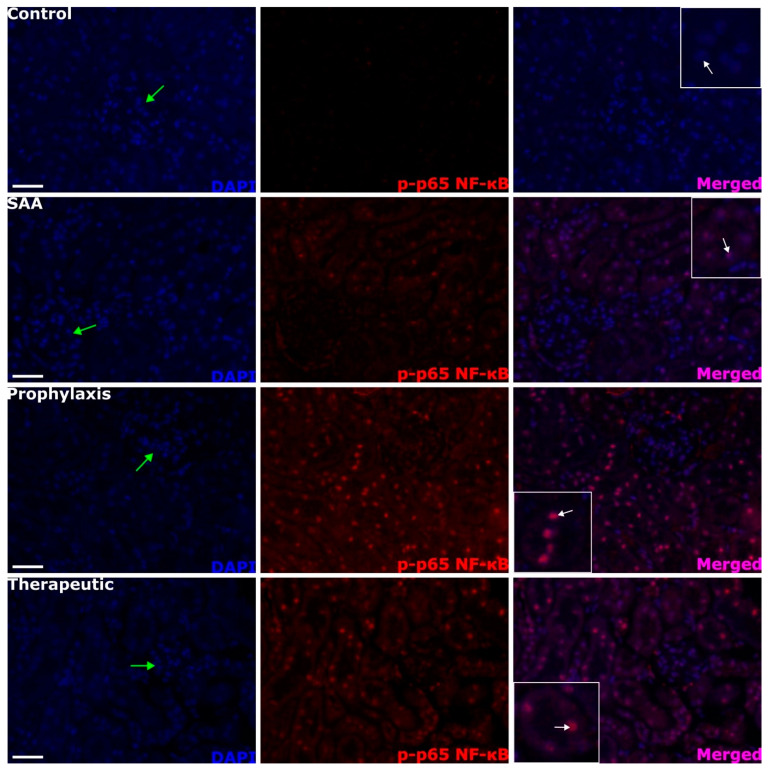
SAA administration stimulates p-p65 NF-κB in renal cortical tissue. Kidney tissue was harvested 4 weeks after the cessation of treatment and fixed in situ before embedding and sectioning (5 µm). Renal sections were dewaxed and then rehydrated before undergoing heat-induced antigen retrieval. Next, p-p65 NF-κB expression was assessed using immunofluorescence microscopy. Slides were visualised at 40× magnification (scale bar = 20 µm); images are representative of at least 4 fields of view for each tissue section from *n* = 6 mice. Nuclei were stained with DAPI (blue) and p-p65 NF-κB with an appropriate opal fluorophore (false coloured red) or merged (purple). Green arrows indicate the location of glomeruli within the renal cortex. Insets show higher-magnification (scale bar = 10 µm) images of renal tubular epithelial cells with NF-kB p-p65+ staining largely co-localised to nuclei (white arrows). Representative images show cortical fields from *n* = 5 (control) and *n* = 6 (SAA) mice. Note, the data pertaining to the control and SAA-treated mice shown in this figure were previously published in a truncated form [32]. The nitroxide intervention was conducted contemporaneously and is now compared to the same published data from the vehicle control and SAA-treated mice.

SAA treatment stimulated the transcriptional activation of NF-κB in the renal cortex, as judged by evaluating phosphorylated p65, which was near absent in the vehicle-treated control mice (c.f., control and SAA panels; Figure 5). The transcriptional activation of NF-κB was again localised primarily to the proximal tubule epithelial cells in SAA-treated mice (compare white arrows in the merged images). The extent and localisation of NF-κB activation were not inhibited by 4-MetT treatment either before or after SAA treatment (c.f., SAA panel with 4-MetT administered as a prophylaxis or therapeutic intervention in Figure 5).

### 2.6. Quantifying Renal Expression of Nrf-2 and p-p65 NFκB after Treatment with SAA in the Presence or Absence of 4-MetT

**Figure 6:**
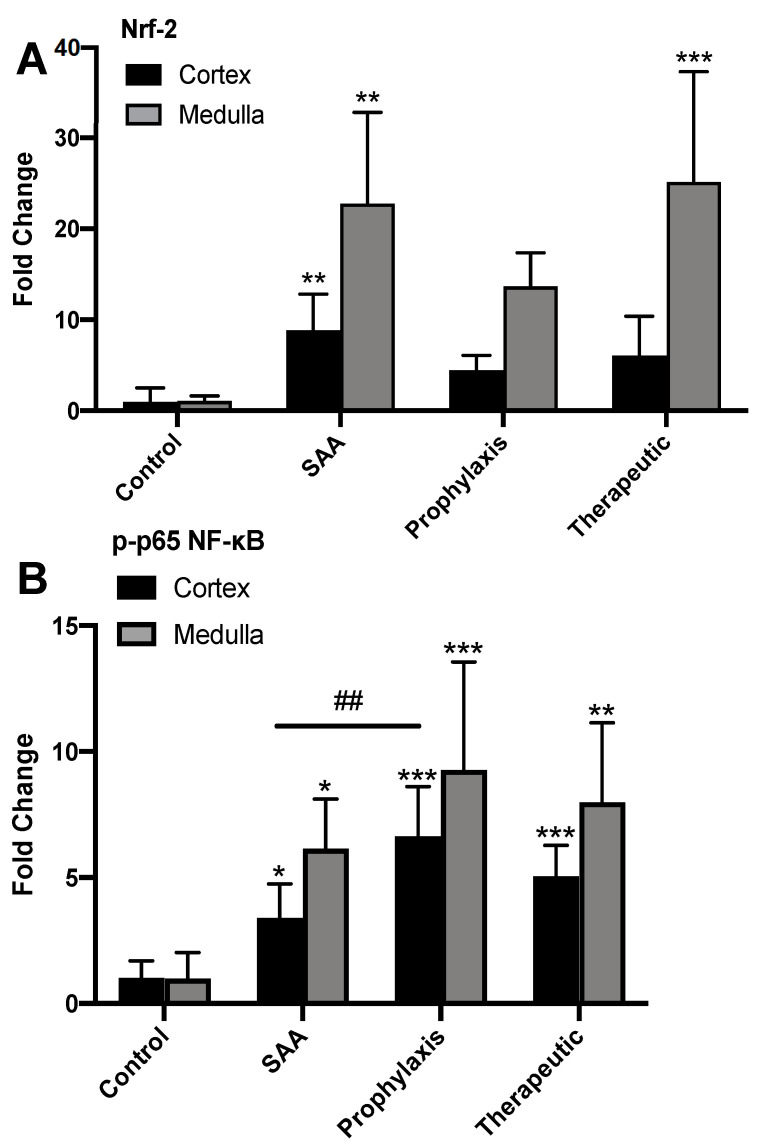
Semi-quantification of renal Nrf-2^+^ and p-p65 NF-κB^+^ immunostaining within the kidney. Four weeks after the cessation of treatment, isolated renal tissues were fixed in situ, embedded, sectioned, and imaged using fluorescent microscopy, as shown in Figure 5 and Figure 6. (**A**) Cortical and medullary Nrf-2^+^ immunostaining was assessed semi-quantitatively through the calculation of mean staining intensity averaged for each sample. (**B**) Cortical and medullary p-p65 NF-κB^+^ immunostaining was assessed semi-quantitatively through calculation of mean staining intensity for each sample. All images were captured at 40× magnification using an immunofluorescence microscope, then averaged for each sample. Data are expressed in terms of relative means ± SD; different relative to the control * *p* ≤ 0.05 ** *p* ≤ 0.01 *** *p* ≤ 0.001; different relative to the SAA group ^##^ *p* ≤ 0.01. Note, the data pertaining to the control and SAA-treated mice shown in this figure were previously published in a truncated form [32]. The nitroxide intervention was conducted contemporaneously and is now compared to the same published data from the vehicle control and SAA-treated mice.

**Figure 7:**
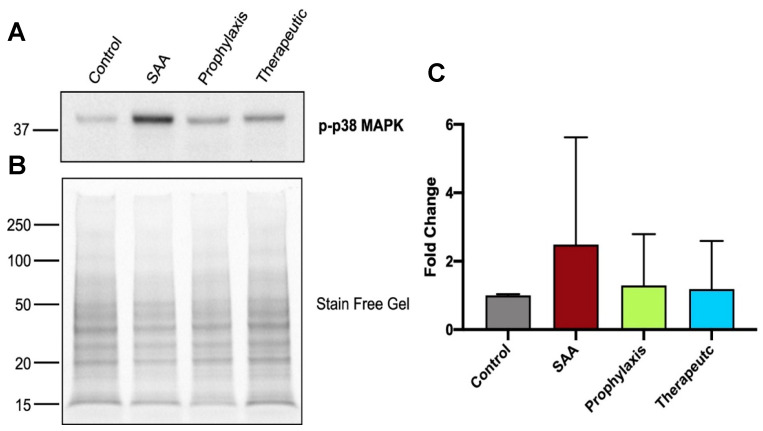
Western blot analyses of renal p-p38 MAPK content. Four weeks after the cessation of treatment, isolated renal tissue was homogenised and the detection and measurement of renal p-p38 MAPK content were performed utilising Western blot analysis. After the separation of protein using SDS-PAGE, proteins were transferred onto a PVDF membrane before incubating with appropriate blocking, washing and antibody preparations. (**A**) Membranes were subsequently imaged with positive bands identified at 38 kDa, corresponding to p-p38 MAPK and quantified using densitometry (ImageLab, version 6.0.1). (**B**) Total protein loading determined from the stain-free gel to enable the normalisation of the p-p38MAPK signal. (**C**) Densitometric data are expressed as mean ± SD. All density data were normalised with total protein loading determined from corresponding stain-free gel images. Data shown represent the fold-change relative to the control. Note, the data pertaining to the control and SAA-treated mice shown in this figure were previously published in a truncated form [32]. The nitroxide intervention was conducted contemporaneously and is now compared to the same published data from the vehicle control and SAA-treated mice.

Semi-quantification of fluorescent intensities (images from Figure 4 and Figure 5) identified that SAA significantly increased the levels of the antioxidant master regulator Nrf-2 in both the renal cortex and medulla, and this outcome remained largely unchanged in the absence or presence of the cyclic nitroxide 4-MetT (Figure 6A). The administration of SAA also markedly increased the level of phosphorylated p65, while the administration of 4-MetT potentiated this signal in the cortex and, to a lesser extent, in the renal medulla (Figure 7B).

### 2.7. SAA Marginally Increases p38MAP-Kinase Activation, Whereas Supplementing with 4-MetT Tended to Diminish This SAA Activity

At 4 weeks after cessation of SAA treatment, the evaluation of SAA-mediated phosphorylation of p38MAPK in renal tissues identified a slight increase in activation compared to the controls, while this increasing trend was not evident in the kidneys from mice treated with 4-MetT (Figure 7A). Semi-quantitation of the Western blotting data showed that while SAA increased the levels of p38MAPK activation, this was not significant, while 4-MetT showed similar levels of p38MAPK activation to the baseline levels in the control.

### 2.8. SAA Stimulates IFN-γ Expression in the Renal Tissue in the Presence or Absence of 4-MetT

**Figure 8:**
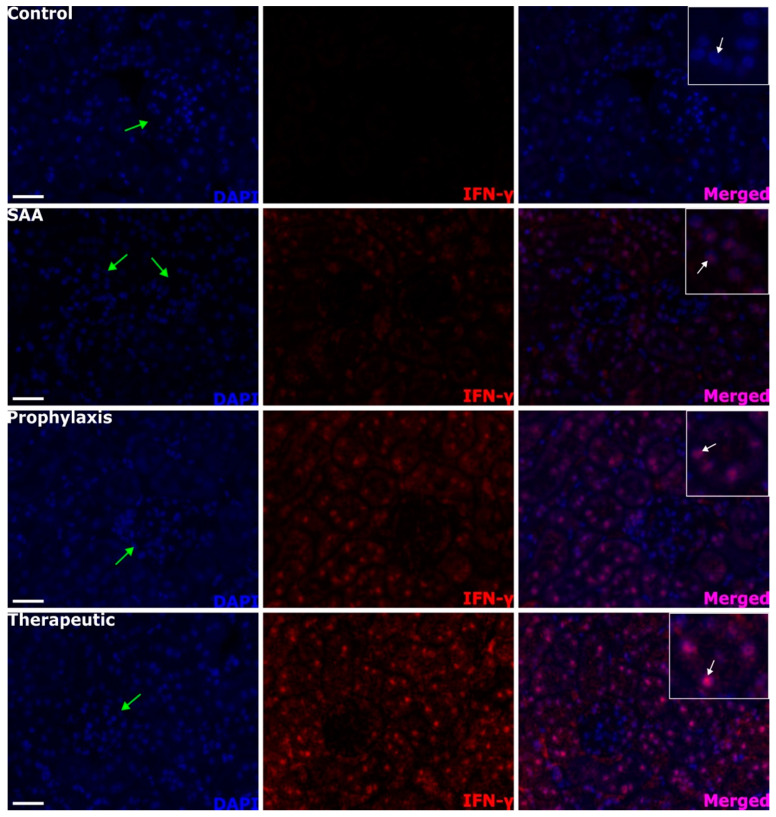
IFN-γ^+^ immunostaining patterns within the renal cortex. Four weeks after the cessation of treatment, isolated renal tissues were fixed in situ, embedded, and sectioned. Patterns of IFN-γ^+^ expression were assessed using immunofluorescence microscopy, with cortical sections being visualised at 40× magnification (scale bar = 20 µm), and at least 4 fields of view were captured for each tissue section from *n* = 6 mice and used for semi-quantitative analyses. Cell nuclei were stained with DAPI (blue) and a relevant fluorophore (red) to evaluate IFN-*γ* expression, or merged (purple). Green arrows indicate the location of glomeruli within the renal cortex, which exhibited low-level IFN-*γ*^+^ immunostaining within the glomerular endothelium. White arrows within the insets highlight high IFN-*γ*^+^ immunostaining localised to the epithelial cells of the proximal tubular network. Note, the data pertaining to the control and SAA-treated mice shown in this figure were previously published in a truncated form [32]. The nitroxide intervention was conducted contemporaneously and is now compared to the same published data from the vehicle control and SAA-treated mice.

SAA treatment stimulated a weak increase in the level of IFN-*γ* in the renal cortex, which was absent in the vehicle-treated control mice (c.f., control and SAA panels in Figure 8). In the presence of both SAA and 4-MetT, the levels of IFN-*γ* increased markedly. Once again, this altered pattern of protein expression was largely localised to proximal tubule epithelial cells in SAA-treated mice (compare the regions identified by white arrows in the merged images). IFN-*γ*^+^ signals were largely absent in cells comprising the glomerulus. The extent and localisation of IFN-*γ* expression were markedly potentiated by 4-MetT treatment when comparing the same regions of the kidney from SAA vs. the intervention treatment groups.

### 2.9. SAA Stimulates Inducible iNOS Expression in the Renal Tissue in the Presence or Absence of 4-MetT

**Figure 9:**
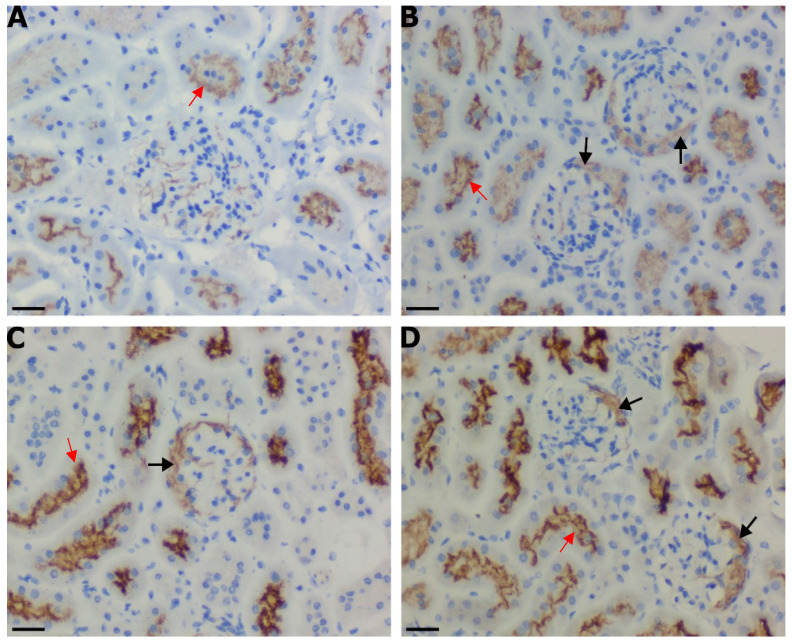
Acute iNOS expression patterns within the renal cortex. Four weeks after the cessation of SAA treatment, isolated renal tissues were fixed in situ, embedded, and sectioned before dewaxing and rehydrating for heat-induced antigen retrieval. The topographical distribution and expression of renal iNOS were evaluated using immunohistochemistry techniques. Cortical sections for the (**A**) control, (**B**) SAA, (**C**) prophylaxis and (**D**) therapeutic groups are visualised at 40× magnification (scale bar = 20 µm) with at least 4 fields of view captured for each section (*n* = 6) for each treatment group before semi-quantitative analyses. Nucleic material was stained with haematoxylin (appearing as blue) and DAB to identify iNOS^+^ expression (appearing as brown). Black arrows highlight brown iNOS^+^ immunostaining localised to the parietal epithelial cells of the renal corpuscle while red arrows indicate iNOS^+^ immunostaining localised to tubule epithelial cells. Note, the data pertaining to the control and SAA-treated mice shown in this figure were previously published in a truncated form [32]. The nitroxide intervention was conducted contemporaneously and is now compared to the same published data from the vehicle control and SAA-treated mice.

The immune protein IFN-γ is known to activate the expression of inducible NOS, among other downstream cellular proteins. To corroborate the presence of SAA-mediated alterations in the IFN-*γ* level in renal tissues, adjacent sections of renal cortex tissue were probed for iNOS expression using an immune-histochemical approach (Figure 9). In kidneys from mice treated with the vehicle (control), a low level of background immune^+^ staining was evident, and this was primarily isolated to the brush-border region of the proximal tubule epithelial cells (red arrows). Treatment with SAA seemed to enhance this expression while also eliciting increased expression in the parietal epithelial cells that define Bowman’s capsule (black arrows). In mice treated with both SAA and 4-MetT, the signal intensity increased again in both the proximal tubule epithelia and the parietal epithelia of Bowman’s capsule, which define the glomeruli.

### 2.10. Quantification of iNOS and IFN-g in the Renal Cortex

**Figure 10:**
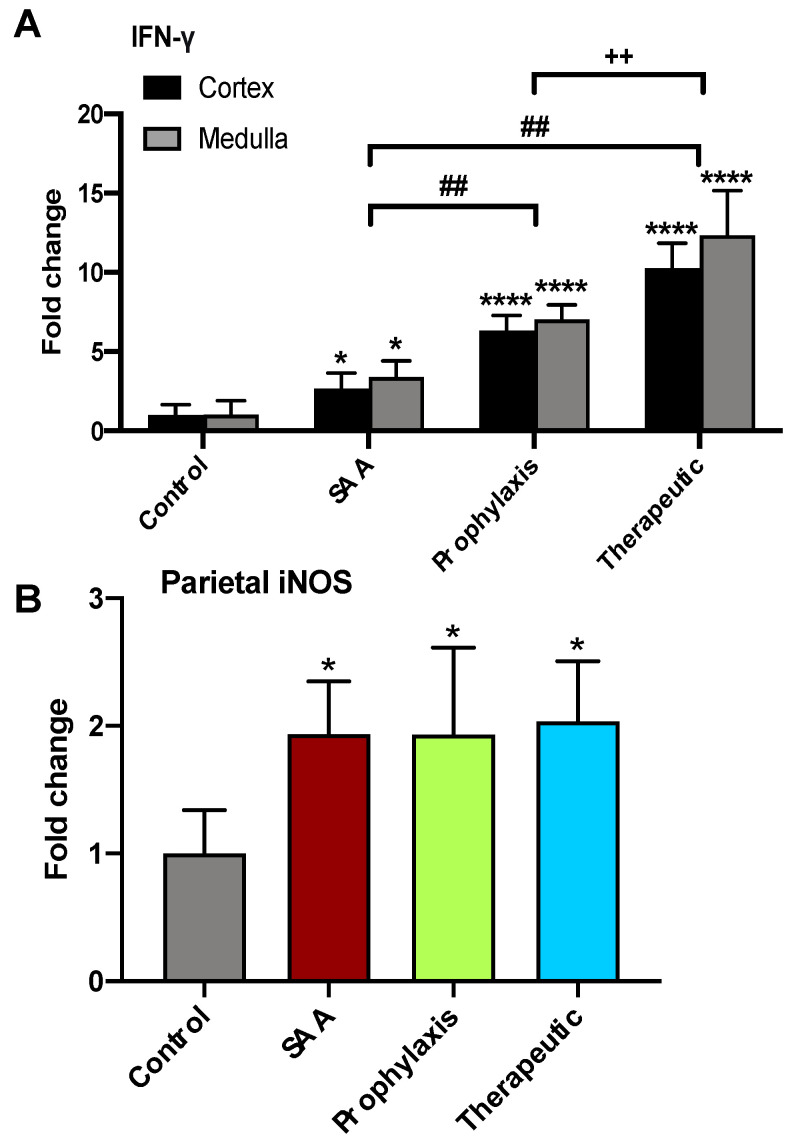
SAA administration stimulates iNOS expression in the renal cortical tissue. Quantification of IFN-gamma and iNOS expression in renal tissues at 4 weeks. (**A**) Cortical and medullary IFN-γ^+^ immunostaining was assessed semi-quantitatively based on representative image capture of the respective regions at 40× magnification using an immunofluorescence microscope, then averaged for each sample. (**B**) Parietal iNOS^+^ immunostaining was assessed semi-quantitatively through the calculation of the mean grey value and optical density calculations based on representative image capture of the respective regions at 40× magnification using light microscopy, then averaged for each sample. Data are expressed in terms of relative means ± SD; significant difference relative to the control * (at least) *p* ≤ 0.05 **** *p* ≤ 0.0001; significant difference relative to the SAA group ^##^ *p* ≤ 0.01; significant difference relative to the prophylaxis group ^++^ *p* ≤ 0.01. Note, the data pertaining to the control and SAA-treated mice shown in this figure were previously published in a truncated form [32]. The nitroxide intervention was conducted contemporaneously and is now compared to the same published data from the vehicle control and SAA-treated mice.

Semi-quantification of immune^+^ staining intensities (images in Figure 8 and Figure 9) identified that SAA significantly increased IFN-*γ* levels in both the renal cortex and the renal medulla, and this outcome was significantly enhanced by the presence of the cyclic nitroxide 4-MetT (Figure 10A). The administered SAA also markedly increased the level of IFN-*γ* co-localising with parietal epithelial cells that defined individual glomeruli. However, the administered 4-MetT showed no effect on iNOS expression (Figure 10B).

### 2.11. SAA Administration Leads to Fibrotic Changes to the Mouse Kidney That Are Ameliorated in the Presence of 4-MetT

**Figure 11:**
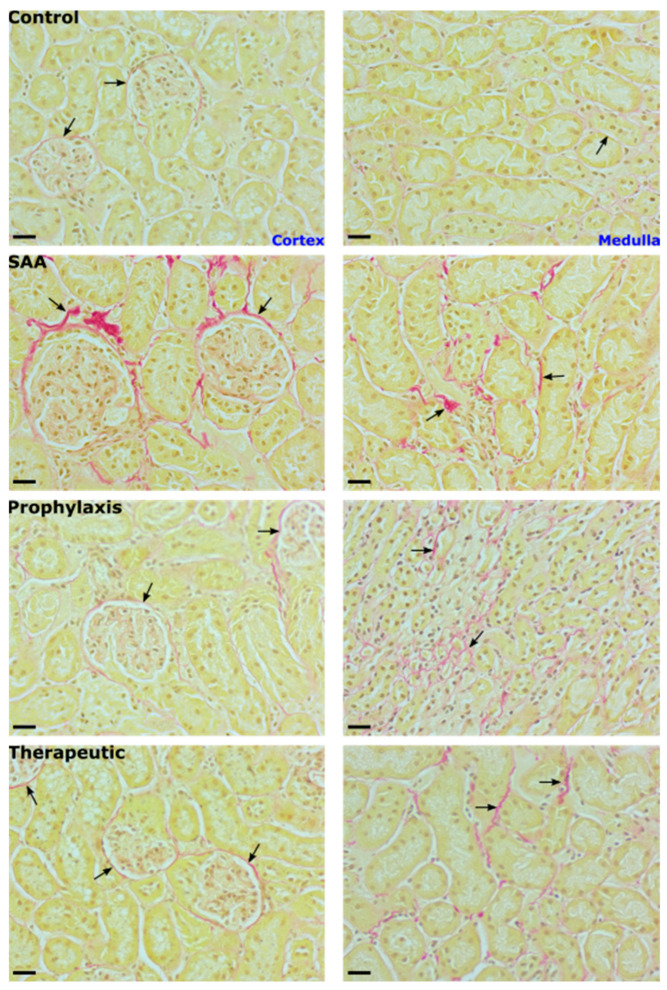
SAA induces enhanced peri-glomerular fibrosis. Sixteen weeks after treatment cessation, renal tissues were fixed in situ, embedded, and sectioned, and the extent of renal fibrosis was evaluated using light microscopy after staining with Picrosirius red. Cortical and medullary renal sections were visualised (40× magnification; scale bar = 20 µm) with at least 4 fields of view captured for each section from *n* = 6 mice in each group. Representative images show nucleic material stained with haematoxylin (brown) and collagen stained with Picrosirius red (red). Black arrows highlight peri-glomerular staining localised to the parietal epithelium and tissue surrounding the glomerular capsule in the renal cortex (left image column) and collagen material within the interstitial and extracellular spaces of the renal medulla (right image column). Note, the data pertaining to the control and SAA-treated mice shown in this figure were previously published in a truncated form [32]. The nitroxide intervention was conducted contemporaneously and is now compared to the same published data from the vehicle control and SAA-treated mice.

Consistent with SAA marginally increasing p38MAP-kinase activity in renal tissues after 4 weeks of monitoring (refer to Figure 7), cortical tissue fibrosis detected 16 weeks after the cessation of treatment also increased in the presence of SAA, as judged by an accumulation of Picrosirius red-stained collagen fibres that was focal to the region around Bowman’s capsule (Figure 11). In the renal medulla, Picrosirius red-stained collagen fibres were present in the interstitial tissue surrounding distil tubules. This positive staining, indicative of the early stages of tissue fibrosis, was not evident in the kidneys from vehicle-treated control mice and was markedly diminished by treatment with 4-MetT (irrespective of whether the nitroxide was administered before or after SAA), suggesting that the supplemented nitroxide decreased p38MAPK activation 4 weeks after the cessation of SAA treatment (Figure 7), which manifested as deceased renal fibrosis (c.f., glomeruli (cortex) and peritubular staining (medulla)) at 16 weeks in the mice supplemented with 4-MetT, irrespective of whether the nitroxide was provided before or after SAA (Figure 11).

### 2.12. SAA Enhances Atherosclerotic Lesion Size While 4-MetT Alters the Thickness and Composition of the Aortic Lesion

**Figure 12:**
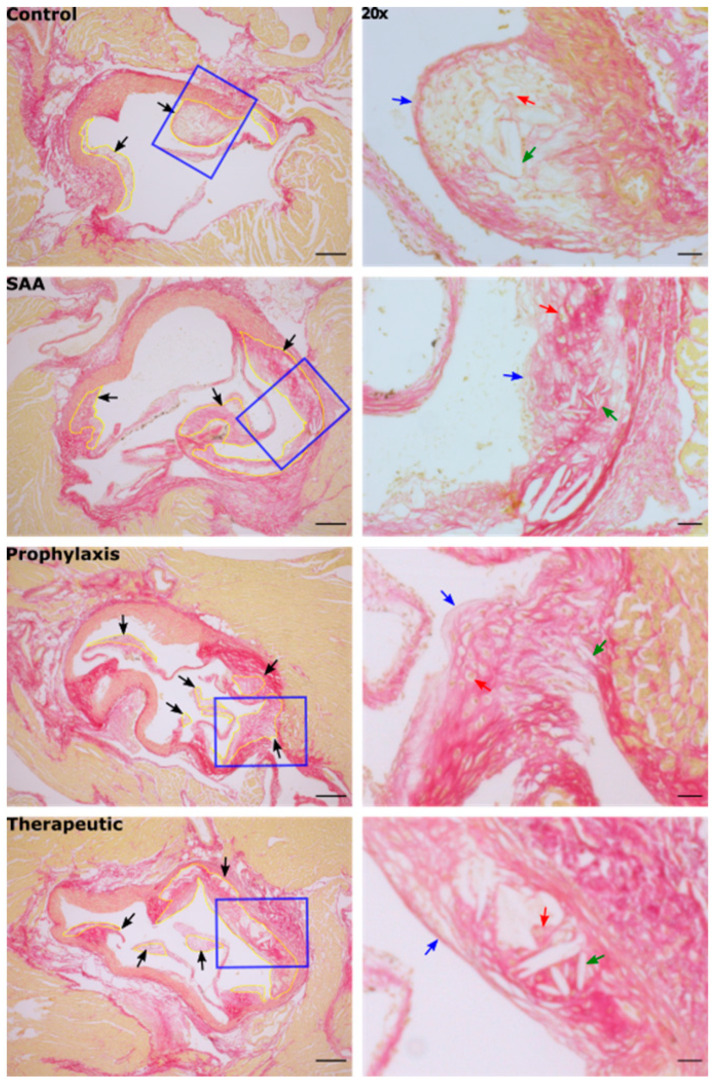
SAA promotes atherosclerotic lesion development at the aortic root leaflet. Sixteen weeks after the cessation of treatment, aortic sinus root tissues were collected, fixed in situ, embedded, and sectioned. The development and progression of aortic tri-leaflet atherosclerosis were evaluated using light microscopy after staining. Aortic root sinus sections were visualised at 5× magnification (scale bar = 200 µm). Cardiomyocyte tissue was stained with haematoxylin (appearing brown) and collagen was stained with Picrosirius red (appearing red). Black arrows with yellow outlines indicate valvular atherosclerotic lesion formation. Blue polygons indicate the field of view magnified at 20×. High magnification views (20× objective, scale bar = 40 µm) highlighting the appearances of the fibrous cap (blue arrow), foam cells (red arrow) and cholesterol clefts (green arrow) associated with the lesion. Note, the data pertaining to the control and SAA-treated mice shown in this figure were previously published in a truncated form [32]. The nitroxide intervention was conducted contemporaneously and is now compared to the same vehicle control and SAA-treated mice.

Stained aortic sections from ApoE^−/−^ from all treatment groups showed the development of complex atherosclerotic lesions at the aortic root, which is the region most prone to atherogenesis in this mouse model [33] (Figure 12).

**Figure 13:**
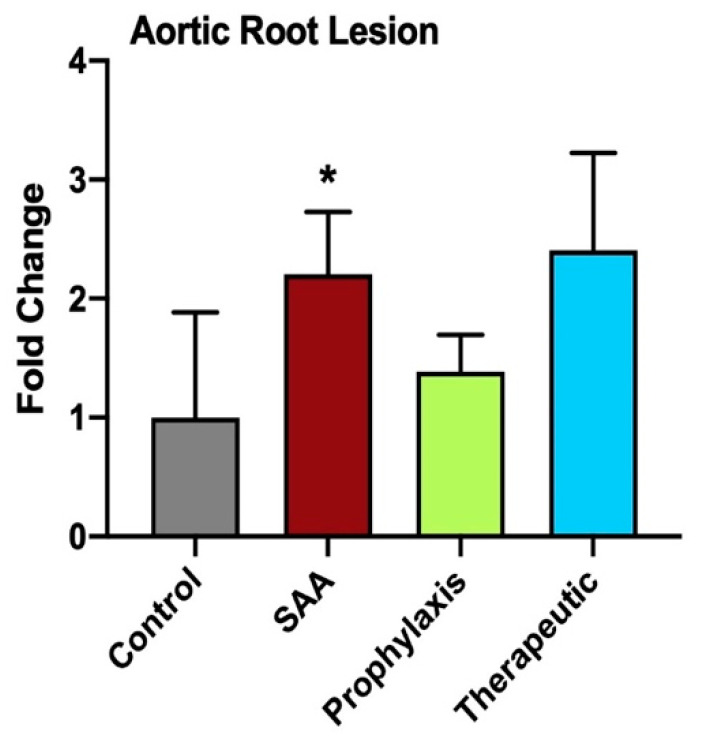
Semi-quantification of tri-leaflet aortic root lesion size at 16 weeks. Panel shows the development and progression of aortic tri-leaflet atherosclerosis evaluated using light microscopy (representative images shown above in Figure 12). The size of the aortic root lesion was evaluated semi-quantitatively using freehand drawing tools (ImageJ, version 2.0) and calculated as a percentage of the corresponding total root area for each individual specimen. Data are expressed in terms of the relative means ± SD; significant difference relative to the control * *p* ≤ 0.05. Note, the data pertaining to the control and SAA-treated mice shown in this figure were previously published in a truncated form [32]. The nitroxide intervention was conducted contemporaneously and is now compared to the same vehicle control and SAA-treated mice.

Consistent with previous research from others that have studied SAA in ApoE^−/−^ mice [34,35,36], vascular lesions in the SAA-treated mice increased significantly in size relative to lesions in the vehicle control (Figure 13), while also producing a diffuse distribution of collagen throughout the lesion compared with the control, which showed a distinctive fibrous cap overlying cholesterol clefts in the core of the lesion (Figure 13). Irrespective of whether 4-MetT was delivered before or after SAA, the total lesion area was not decreased significantly compared with the SAA group (Figure 13).

## 3. Discussion

The precise role that circulating SAA plays in stimulating inflammatory cascades in both acute and chronic inflammatory disease remains unclear, with evidence of different SAA isoforms linked to disease progression [8]. In this study, the provision of exogenous recombinant SAA (a consensus molecule of the SAA1 and 2 isoforms) to elicit an acute-phase response in ApoE^−/−^ mice led to an accumulation of inflammatory markers in the kidney, which subsequently manifested in long-term alterations to renal tissue and the aortic root, synonymous with renal dysfunction and atherosclerotic lesion formation, respectively. The upregulation of the oxidative stress-related transcription factors Nrf-2 and *p*-p65 NF-κB and the subsequent downstream phosphorylative activation of p38MAPK led to a concerted immune response characterised by increases in IFN-*γ* and iNOS proteins, which culminated in the endpoints of accelerated renal fibrosis and enhanced atherosclerotic lesion formation at the aortic root relative to the vehicle-treated control mice. Phosphorylative activation of p38MAPK in renal tubular epithelia in the presence of SAA has been reported previously [37]. Accordingly, in SAA-treated mice, *p*-p38 MAPK was detected with a nuclear localisation in the tubular epithelial cells that line both proximal and distil tubules, representing a similar distribution to that determined for Nrf-2 and NF-κB following the exogenous SAA induction phase. Consequently, this combination of SAA pro-inflammatory activity manifested as increased renal fibrosis that was focal to the external surface of Bowman’s capsule and distributed in a peritubular fashion (affecting both proximal and distil tubules) when assessed 16 weeks after the cessation of SAA administration.

The therapeutic potential and anti-fibrotic properties of cyclical nitroxides remain elusive, with conflicting findings reported by various researchers. Minoxidil, a nitroxide radical currently prescribed as an anti-hypertensive agent, can attenuate collagen cross-linking by inhibiting lysyl hydroxylase in both in vitro and in vivo settings [38,39], highlighting the potential of nitroxides to act as anti-fibrotic drugs. On the contrary, Zuurmond et al. reported that minoxidil’s inhibition of lysyl hydroxylase has little effect in total level of hydroxyallysine cross-linking [40], an essential process during collagen modification and the formation of an atherosclerotic fibrous cap [41]. In the current study, the intervention with the cyclic nitroxide 4-MetT supplemented before (prophylaxis) or after (therapeutic) SAA stimulation in mice was generally unable to inhibit SAA pro-inflammatory activity in the short term; however, in the longer term, nitroxide supplementation ameliorated renal fibrosis and facilitated some reorganisation of atherosclerotic plaques to yield a higher content of organised collagen networks, especially underlying the fibrous cap of the developed lesion. Therefore, although the nitroxide tested here lacked specific pharmacological activity against SAA-mediated acute inflammation, the anti-fibrotic action in the kidney combined with the altered collagen network in the atherosclerotic lesions are consistent with 4-MetT protecting renal tissues and potentially stabilising vascular lesions. Additionally, in the context of atherosclerosis, the findings from this study suggest that 4-MetT-mediated collagen network reorganisation facilitates the formation of a more stable plaque, which may be less prone to rupture and hence less likely to cause subsequent myocardial infarction or stroke [42,43]. However, the therapeutic potential of 4-MetT and other cyclic nitroxides in this role as anti-fibrotic drugs warrants further investigation.

The potential for interplay between the transcription factors Nrf-2 and NF-κB, and how this interaction can modulate cellular responses induced by acute phase SAA, is unclear. In this study, both *p*-p65 NF-κB- and Nrf-2-positive immunostaining was predominantly localised to the nuclei of renal tubular epithelia (within the proximal and distal tubules), suggesting that the transcriptional activation of both proteins is a consequence of targeted SAA bioactivity. Furthermore, the fact that both Nrf-2 and NF-κB transcription factors were simultaneously upregulated within the renal tissue indicates the potential for a cooperative relationship following SAA’s induction of renal inflammation. Accordingly, the results from this study suggest that SAA administration causes a redox imbalance leading to oxidative stress specifically within the tubular epithelial cells and parietal epithelial cells that line Bowman’s capsule, rather than the other cell types within the glomeruli. Paradoxically, when co-supplemented with the cyclic nitroxide 4-MetT, which mimics antioxidant SOD activity, both the expression and localisation of the master regulator Nrf-2 remained unchanged, consistent with the idea that oxidative stress alone does not drive Nrf-2/NF-κB transcriptional activation.

The pro-inflammatory cytokine IFN-*γ* is also linked to immunomodulation in the kidney [44]. In the present study, IFN-*γ*^+^ immune-staining was localised predominantly to the nuclei of renal tubular epithelial cells, with minor glomerular localisation specifically in the parietal epithelial cells that form a uniform monolayer on the inner surface of Bowman’s capsule. IFN-*γ* can upregulate proteins involved in the production of reactive oxygen (e.g., NADPH oxidases [45]) and nitrogen species (e.g., iNOS-mediated increase in peroxynitrite [46,47]), thereby propagating oxidative and nitrosative stress responses in kidneys. In this sense, the acute renal response to SAA may evoke pathways which amplify and sustain large oxidative/nitrosative imbalances, and this may explain why the activation of Nrf-2 and NF-κB (both redox sensitive transcription factors) is sustained even when the antioxidant 4-MetT is co-supplemented with SAA.

It is now established that impaired kidney function can facilitate systemic endothelial dysfunction, which manifests as early-stage atherosclerotic lesion development [48,49,50]. Several studies have identified that SAA enhances atherosclerotic lesion formation in conjunction with eliciting parallel renal dysfunction [34,37,51]. Also, chronic kidney disease induces systemic inflammation, potentially exacerbating the process of atherosclerosis [52], thereby providing an avenue to explain how SAA-mediated kidney damage can promote vascular plaque formation. Furthermore, our data demonstrating a direct impact of SAA on acetylcholine-mediated vascular relaxation suggest that SAA bioactivity causes vascular dysfunction as an early event linked to atherogenesis. Accordingly, the atherosclerotic lesion area was significantly greater in the SAA group compared to the vehicle-treated control group. Atherosclerotic lesion complexity has also shown been to be a relatively important prognostic criterion compared to the extent of total lesion size [53]. In this study, the histopathologic features of vascular lesions stimulated by SAA included a relatively higher density of cholesterol clefts and a lack a clear peripheral margin due to the lower density of cells forming the cap combined with a more diffuse network of collagen fibres, which are both considered to be signs of clinically unstable lesion development [43]. Further intervention studies utilising cyclic nitroxides in plaque rupture models would be required to corroborate these findings and demonstrate plaque stabilisation.

In contrast, the vascular lesions in the vehicle-treated control group showed a relatively thin well-defined collagen-containing fibrous cap (Figure 12). Therefore, SAA not only accelerated aortic lesion formation, but the lesion architecture and the diffuse fibrous network likely represent a worse prognosis for acute events, and thus may highlight a need to prevent inappropriate SAA bioactivity in vivo following the acute phase reaction (or a series of such acute phase reactions). In this context, the supplemented nitroxide 4-MetT tested here, although unable to decrease the aortic lesion size, was able to facilitate a subtle reorganisation of the intraplaque collagen network, thereby providing a more uniform fibrous distribution synonymous with a more stable atherosclerotic lesion.

A limitation of this study is reflected by the additional acute-phase isoform SAA3 expressed in mice, which is only weakly associated with HDL in mice. Therefore, SAA3 may exacerbate the pro-inflammatory state induced by exogenous SAA administration in mice, but not in humans. While this may not accurately encapsulate SAA pathophysiology in humans, it may help to explain the absence of improvement in SAA-induced renal dysfunction and atherosclerosis with 4-MetT supplementation. Furthermore, the relatively low number of samples per group decreased the statistical power, which resulted in some trends not reaching the requirement for significance.

## 4. Methods and Materials

### 4.1. Vascular Function Studies

#### 4.1.1. Animals

Freshly isolated aortic segments were obtained from male Wistar rats (Animal Resource Centre, Perth, Western Australia) with local ethics approval (AEC approval 2010/007). Where required, animals were anaesthetised (3% *v*/*v* isofluorane gas/oxygen), and a thoracotomy was performed to expose the heart, which was gravity-perfused with phosphate-buffered saline (pH 7.4; 150 mM NaCl) directly into the left ventricle. Flushed aortae were harvested and immersed in a Krebs–Henseleit solution.

#### 4.1.2. Vascular Reactivity

The vascular reactivity of isolated aortae was examined using a previously established protocol [54]. Briefly, isolated aortae were placed in a modified Krebs–Henseleit solution (containing in mM: 11 D-glucose, 1.2 MgSO_4_, 12 KH_2_PO_4_, 4.7 KCl, 120 NaCl, 25 NaHCO_3_, and 2.5 CaCl_2_·2H_2_O) and cut into 5 mm rings. Aortic rings were mounted in a Myobath system (World Precision Instruments, Inc., Sarasota, FL, USA) containing 15 mL of modified Krebs–Henseleit solution aerated at 37 °C with 5% *v*/*v* CO_2_(g) and contracted with phenylepherine (dose 10^−9^–10^−5^ mol/L) at the dose that caused half-maximal contraction in each ring. Next, mounted rings were pre-treated with the vehicle (control); L-NAME (negative control); SAA (final 10 μg/mL); or SAA + added 4-MetT (final concentrations 12.5 or 25 μM) for 4 h followed by washing, pre-constriction, and relaxation. Concentration–response relaxation curves (10^−9^–10^−5^ mol/L) to an endothelium-dependent vaso-stimulator (acetylcholine; ACh) were constructed in the presence of indomethacin (25 μM) and relaxation expressed as the percentage of the initial contraction of the individual aortic rings.

### 4.2. Murine Model

All studies involving mice were conducted with appropriate ethics approval (AEC approval 2018/1408). Male ApoE^−/−^ mice (Animal Resource Centre, Perth, WA, Australia) were acclimated for one week at the Charles Perkins Centre Animal Facility prior to study commencement. Mice were randomly housed in groups of 6 animals per cage (12 h light–dark cycle at 22 °C) with a standard chow diet (cat#23200-12152, Specialty Feeds, Glen Forrest, WA 6071) and water provided ad libitum throughout the study. Mice were not fed a high-fat diet in these studies intentionally, as an increase in lipid load may stimulate a pro-inflammatory state that overwhelms any subtle impact of supplemented recombinant SAA. Intervention studies with 4-MetT were performed concurrently with mice studied in the absence (vehicle control) or presence of recombinant SAA (PeproTech, Cranbury, NJ, USA), which represents a consensus molecule of the human SAA1 and 2 isoforms. Outcomes from studying the control and SAA-treated mice in this cohort were reported separately in Ref. [32] as part of the first author Gao’s master’s degree in Medicine. Subsequently, Gao enrolled in a postgraduate medical degree while completing his master’s degree concurrently, which delayed the full analysis of the archived intervention studies with 4-MetT in comparison with the same vehicle control and SAA-treated groups reported earlier. Thus, although the control, SAA-stimulus, and intervention studies with 4-MetT were all conducted simultaneously under the same experimental conditions, we now report on the interventional outcomes relative to the previously published control and SAA-stimulus data.

### 4.3. Experimental Design

Male ApoE^−/−^ mice (8 weeks of age) were randomly allocated into 4 groups each containing 12 mice and assigned as described below.

(i) Vehicle control: ApoE^−/−^ mice were administered 100 µL of phosphate-buffered saline (PBS) via intraperitoneal (i.p.) injection every 3 days for 14 days.
(ii) SAA group: ApoE^−/−^ mice were administered 100 µL of filter-sterilised recombinant human SAA (120 µg/mL) via i.p. injection every 3 days for 14 days. This dose was determined to be equivalent to a short-term moderate level of SAA within the circulation which can further rise in an acute-phase response.
(iii) Prophylaxis group: ApoE^−/−^ mice were administered 100 µL of 4-MetT (15 mg/kg) via i.p. injection daily for 2 weeks followed by 100 µL of SAA (as described above).
(iv) Therapeutic group: ApoE^−/−^ mice were administered 100 µL of SAA (as above) followed by 100 µL of 4-MetT (15 mg/kg) via i.p. injection daily for 2 weeks.

Experimental groups of mice (*n* = 6) were then analysed at 4 or 16 weeks after the cessation of SAA or 4-MetT treatments where tissue samples were harvested, assigned for subsequent biochemical and histological analyses and processed as we described previously [32]. These time points were selected to represent the earliest stages of atherogenesis followed by a long-term endpoint for more substantial pathological change to the vasculature allowing for an evaluation of the intervention with cyclic nitroxides reported here.

### 4.4. Live Animal Imaging

To assess MMP activity in situ, mice were administered a commercial MMPsense probe (2 nM; 150 µL) via tail vein injection 4 weeks after SAA stimulation, and then unconscious mice were imaged with an IVIS^®^ SpectrumCT (PerkinElmer, Waltham, MA, USA) as detailed elsewhere [32]. Qualitative MMP sense signals generated in vivo were evaluated using Living Image^®^ (PerkinElmer) standard analysis software (v 4.5). After imaging, mice were euthanised, their kidneys were isolated, and then were immediately re-imaged, and the resultant signals were quantified to compare in situ and ex vivo MMP sense signals in the same kidneys.

### 4.5. Urine Collection

Urine samples were collected from mice prior to deep anaesthesia, snap-frozen and stored at −80 °C until required for biochemical analyses, as described elsewhere [32].

### 4.6. Collection of Tissue Specimens

Upon excision, some kidneys were assigned to histological analyses and were fixed ex vivo in 4% *v*/*v* formalin and subsequently embedded in paraffin. Kidney samples were stored as paraffin blocks (22 °C in the dark) until required. For biochemical analyses, other samples of kidney and aortae were snap-frozen in liquid nitrogen and stored at −80 °C for subsequent tissue homogenisation (below). Where required, embedded kidney tissues were sectioned (5 μm), transferred to Superfrost Plus^TM^ slides (Thermo Fisher Scientific, Scoresby, Victoria, Australia), and then dewaxed and rehydrated. Sections probed with immunohistochemistry or immunofluorescence were imaged using Zen 2 Lite software (Carl Zeiss, Sydney, Australia). Image analyses and other image-based measurements were performed with Image J software (v.2.0, NIH, Bethesda, MD, USA).

### 4.7. Tissue Homogenisation

Mouse kidneys or rat aortic rings (from vascular function studies) were thawed, cut into small pieces, snap-frozen in liquid N_2_, and pulverised with a mortar and pestle. Next the tissue was warmed to 22 °C and suspended in lysis buffer (50 mM PBS, pH 7.4 with 1 mM EDTA, 10 μM butylated hydroxytoluene and a Protease Inhibitor Cocktail tablet (Roche Diagnostics, Bern, Switzerland)) and then homogenised for 5 min using a rotating piston (Wheaton glass, Millville, NJ, USA). All homogenates were centrifuged (2500× *g*) and the clarified supernatant stored at −80 °C for biochemical analysis. To account for variation in tissue wet weight, total homogenate protein (assessed using the bicinchoninic acid (BCA), Sigma-Aldrich, Sydney, Australia) was used to normalise all biochemical parameters.

### 4.8. Biochemical Assays

#### 4.8.1. Vasodilating cGMP

The vasoactive molecule cGMP was measured in homogenised rat aortae after the completion of relaxation studies using an ELISA kit (Enzo, Farmingdale, NY, USA). Both serially diluted standards at 100–0.16 pmol/mL and clarified homogenates were acetylated according to the manufacturer’s instructions and samples were read at 405 nm using an Infinite M1000 plate reader (Tecan, Männedorf, Switzerland) displayed on Tecan i-control software (v 200 Pro). Next, the levels of aortic cGMP were interpolated from the standard curve generated in parallel. Protein-normalised aortic cGMP values were then calculated and averaged for each corresponding series of aortic rings in the same treatment group.

#### 4.8.2. Biomarkers of Renal Injury

The expression levels of kidney injury molecule-1 (KIM-1) and total urinary protein were determined using commercial kits and mouse urine samples as described in detail elsewhere [32].

#### 4.8.3. Interferon-Gamma (IFN-*γ*)

Renal homogenate levels of the cytokine IFN-*γ* were determined using a commercial ELISA kit (elisakit.com, Australia), following the manufacturer’s instructions.

#### 4.8.4. Western Blot Studies for p-p38 MAPK

Levels of phosphorylated p38MAPK in mouse kidney homogenates were semi-quantified with Western blotting using an anti-phospho-p38MAPK antibody (Cell Signaling Technologies, Danvers, MA, USA) as described in [32]. Membranes were imaged using a ChemiDoc Touch system (BioRad, Sydney, Australia) and densitometric analysis conducted with ImageLab^™^ software (v 6.0.1, 2017, BioRad, Sydney, Australia). All data were normalised against total protein (bands detected in situ) using the corresponding membranes. Changes in p38MAPK activation were expressed relative to the control.

### 4.9. Immunofluorescence Studies

Deparaffinised and rehydrated 5 μm kidney sections underwent antigen retrieval and then were probed with a primary antibody (from the following: anti-phospho-NF-κB p65, Sigma-Aldrich, dilution 1:100 *v*/*v*; anti-Nrf2, Abcam, dilution 1:700 *v*/*v*; anti-IFN-*γ*, Abcam, dilution 1:3000 *v*/*v*; anti-phospho-p38 MAPK, Cell Signaling Technologies, dilution 1:800 *v*/*v*) followed by appropriate fluorescent secondary antibodies, and then were subjected to imaging with a Zeiss Axioscope A1 fluorescent microscope (Zeiss, Sydney, Australia) [32]. Random fields of view were imaged from both the cortical and medullary regions and converted to the JPEG format for subsequent image analyses with Image J (v.2.0, NIH).

#### 4.9.1. Immunohistochemistry Studies

To assess the level and tissue distribution of iNOS, sections of mouse kidney tissue were incubated with polyclonal anti-iNOS primary antibody (final dilution 1:200 *v*/*v*; Abcam, Melbourne, Australia) followed by a ready-to-use horseradish peroxidase (HRP)-labelled polymer conjugated secondary antibody (DAKO, Sydney, Australia) before cover slipping and imaging with a Zeiss Axioscope A1 light microscope fitted with a digital camera (Carl Zeiss, Sydney, Australia), as described previously [32]. Random fields of view were imaged from both the cortical and medullary regions and converted to the JPEG format for image analyses with Image J (v.2.0, NIH).

#### 4.9.2. Assessing Tissue Fibrosis with Picro-Sirius Red Staining

Sections of kidney and aortic root were prepared from isolated organs taken from mice 16 weeks post cessation of SAA treatment and were used to assess fibrotic changes and lesion size and composition after Picrosirius red staining, as described in [32]. Slides were imaged with Zeiss Axioscope A1 light microscope fitted with a digital camera (Carl Zeiss, Sydney, Australia). Random fields of view were imaged from both the cortical and medullary regions (kidney), or the aortic root (lesion), then were converted to the JPEG for the evaluation of Picrosirius red staining intensity (Image J v.2.0, NIH). The percentage of lesion area was calculated at 5× magnification by outlining each lesion area that was then summed to give a total area, which was then normalised to the total area of the corresponding aortic root. Lesion histological analyses were conducted at 20× magnification.

### 4.10. Statistical Analysis

Statistical analysis was performed using GraphPad^®^ Prism Version 8.0 (GraphPad Software Inc., La Jolla, CA, USA). All group data were initially tested for normalcy (Shapiro–Wilk and Kolmogorov–Smirnov tests) and, where appropriate, between group differences were compared using parametric one-way ANOVA with Tukey’s post hoc test. Data were expressed as relative means ± standard deviation (SD). Differences between mean data were taken to be statistically significant at a 95% confidence interval (*p* < 0.05).

## 5. Conclusions

Combined, the data from this study corroborate that SAA induces an array of inflammatory cascades that can underpin both renal and vascular dysfunction. Accordingly, transient increases in the acute-phase protein SAA stimulates multiple molecular responses that include bolstering antioxidant elements via the master regulator Nrf-2 (a direct response to oxidative stress) and the induction of pro-inflammatory and pro-fibrotic pathways that manifest as both renal and vascular dysfunction in the short term (at 4 weeks) and renal fibrosis and potentiated atherosclerosis in the longer term (at 16 weeks). This complexity of SAA signalling combined with the capacity of this acute-phase protein to amplify pro-inflammatory molecular pathways in immune cells leading to tissue fibrosis following the acute response requires careful consideration when developing therapeutic drugs to combat SAA. As such, therapeutically targeting SAA-induced cellular activation will likely require the development of a drug (or a combination of drugs) that inhibits multiple pathways to garner an optimal therapeutic advantage. Considering these criteria in the design of an effective SAA inhibitor, it is concluded that the cyclic nitroxide 4-MetT was largely unable to directly ameliorate the multiple biological activities of SAA. Overall, a direct link between 4-MetT and the inhibition of SAA bioactivity cannot be drawn based on the evidence provided here. However, 4-MetT was able to inhibit renal fibrosis (possibly linked to inhibiting p38MAP-kinase activity) and alter the distribution of the collagenous fibre network in the developing atherosclerotic plaque in this animal model. This indicates that future work is warranted to evaluate the anti-fibrotic effects of cyclic nitroxides in other inflammatory states, although the usefulness of this class of drug as an inhibitor of SAA bioactivity may be limited.

## Data Availability

The original contributions presented in the study are included in the article; further inquiries can be directed to the corresponding author to make available primary data.
